# Real-Time Neuropsychological Testing for Hydrocephalus: Ultra-Fast Neuropsychological Testing During Infusion and Tap Test in Patients with Idiopathic Normal-Pressure Hydrocephalus

**DOI:** 10.3390/brainsci15010036

**Published:** 2025-01-01

**Authors:** Ilaria Guarracino, Sara Fabbro, Daniele Piccolo, Serena D’Agostini, Miran Skrap, Enrico Belgrado, Marco Vindigni, Francesco Tuniz, Barbara Tomasino

**Affiliations:** 1 Scientific Institute, IRCCS E. Medea, Dipartimento/Unità Operativa Pasian di Prato, 33037 Pasian di Prato, Italy; ilaria.guarracino@lanostrafamiglia.it; 2 Neurosurgery Unit, Head-Neck and Neurosciences Department, Santa Maria della Misericordia University Hospital, 33100 Udine, Italy; sara.fabbro@asufc.sanita.fvg.it (S.F.); daniele.piccolo@asufc.sanita.fvg.it (D.P.); miran.skrap@gmail.com (M.S.); marco.vindigni@asufc.sanita.fvg.it (M.V.); 3 Neuroradiology Unit, Department of Diagnostic Imaging, Santa Maria della Misericordia University Hospital, 33100 Udine, Italy; serena.dagostini@asufc.sanita.fvg.it; 4 Department of Neurology, Santa Maria della Misericordia University Hospital, 33100 Udine, Italy; enrico.belgrado@asufc.sanita.fvg.it

**Keywords:** neuropsychology, real time, idiopathic hydrocephalus, cerebrospinal fluid

## Abstract

Background/Objectives: Ventriculoperitoneal shunting is a validated procedure for the treatment of idiopathic normal-pressure hydrocephalus. To select shunt-responsive patients, infusion and tap tests can be used. Only gait is evaluated after the procedure to establish a potential improvement. In this study, we present our Hydro-Real-Time Neuropsychological Testing protocol to assess the feasibility of performing an ultra-fast assessment of patients during the infusion and tap test. Methods: We tested 57 patients during the infusion and tap test to obtain real-time feedback on their cognitive status. Data were obtained immediately before the infusion phase (T0), when the pressure plateau was reached (T1), and immediately after cerebrospinal fluid subtraction (T2). Based on cerebrospinal fluid dynamics, 63.15% of the patients presented a resistance to outflow > 12 mmHg/mL/min, while 88% had a positive tap test response. Results: Compared to T0, cerebrospinal fluid removal significantly improved performance on tasks exploring executive functions (counting backward, *p* < 0.001; verbal fluency, *p* < 0.001). Patients were significantly faster at counting backward at T2 vs. T1 (*p* < 0.05) and at T2 vs. T0 (*p* < 0.001) and were significantly faster at counting forward at T2 vs. T1 (*p* < 0.005), suggesting an improvement in speed at T2. There was a significantly smaller index at T1 vs. T0 (*p* = 0.005) and at T2 vs. T0 (*p* < 0.001), suggesting a more marked improvement in patients’ executive abilities at T2 and a smaller improvement at T1. Regarding verbal fluency, patients were worse at T1 vs. T0 (*p* < 0.001) and at T2 vs. T0 (*p* < 0.001). Conclusions: Patients’ performance can be monitored during the infusion and tap test as significant changes in executive functions are observable. In future, this protocol might help improve patients’ selection for surgery.

## 1. Introduction

Idiopathic normal-pressure hydrocephalus (iNPH) is characterized by ventricular dilatation due to a disturbance in cerebrospinal fluid dynamics, excessive cerebrospinal fluid (CSF) accumulation, and normal intracranial pressure [1]. Clinical signs of iNPH are characterized as a triad [2]: (i) slow and progressive gait and balance deficits, (ii) cognitive decline, and (iii) urinary incontinence [3]. The treatment of iNPH involves a ventriculoperitoneal shunt [4,5,6,7,8]. The present study aims to fill the gap in the information collected during the infusion and tap test [9,10,11]. Until now, only a walking test has been performed to evaluate clinical improvement in patients with suspected normal-pressure hydrocephalus and to select individuals who may benefit from a ventriculoperitoneal shunt. The patient’s neuropsychological status (cognitive decline is one-third of the triad symptoms [12,13,14]) is unfortunately not assessed. It is generally held that better methods for the identification of responders and non-responders are needed. To date, cognitive status has been addressed only before or after the tap test [15,16,17]. Pre- and post-tap test cognitive assessments [18] showed, for instance, that a 5-point increase in MOCA scores is matched by an improvement in cognitive domains; an MMSE increase of at least three points can be considered a positive predictor for a shunt intervention [17]; and an amelioration between 30 and 60 min after the tap test can be found in identical forms, Bingley’s memory test, and the reaction time test [15]. Improvements in several tests were also reported after shunt surgery, highlighting the predictive value of cognitive assessment [16]. Undoubtedly, pre- and post-tap test cognitive assessments remain a standard source of information; however, the procedure could also be improved by adding complementary data on the patient’s cognitive status. Studies have attempted to predict cognitive improvement post-ventriculoperitoneal shunt solely based on pre- or post-tap testing cognitive scores. If it is proven feasible, the intra-tap test cognitive assessment—occurring when pressure is manipulated—could, in principle, be a direct measure for predicting cognitive changes post-ventriculoperitoneal shunt. In the present study, we addressed patients’ cognitive performance during the procedure, in order to assess the feasibility of ultra-fast neuropsychological testing during CSF pressure modification. Based on our experience with Real-Time Neuropsychological Testing (RTNT) in awake surgery [19], we performed a Hydro-RTNT in an iNPH setting. The neuropsychological protocol was administered at the patient’s bedside, immediately before the infusion test (T0), when a stable pressure plateau was reached (T1), and immediately after CSF subtraction at the end of the tap test (T2). We hypothesized that fluctuations in cognitive performance between T0 and T1 (at the end of the infusion phase, when the intracranial pressure was maximal) would indicate that changes can be observed in real time in patients with iNPH owing to the increase in intracranial pressure. Thus, the cognitive aspect may become a relevant evaluation index in future tap test responders. Furthermore, possible fluctuations in cognitive performance between T0 and T2 (after CSF subtraction, when the intracranial pressure reached values near 0 mmHg) would suggest that there may be improvements due to CSF drainage during the tap test.

## 2. Materials and Methods

### 2.1. Patients

Patients meeting the following criteria were included in this study:

- Subjects with iNPH eligible for the CSF Tap Test and infusion test studied by the Department of Neurosurgery according to criteria based on the iNPH guidelines [20,21]. At our neurosurgery department, the CSF Tap Test is used to identify subjects with higher chances of response to surgical treatment [22].
- Patients aged 60 to 90 years.
- Patients who gave their informed consent to participate in the study.
- Patients meeting at least one of the following criteria were excluded from the study:
- Non-native Italian speakers (language proficiency is required to perform neuropsychological tests, including language functions);
- Known acquired structural abnormalities of the central nervous system, such as outcomes of ischemic events, traumatic events, or outcomes of neurosurgical interventions;
- Known congenital structural abnormalities of the central nervous system, such as cerebral or cerebrovascular malformations;
- Known neurocognitive developmental deficits;
- Other types of hydrocephalus, i.e., triventricular, neonatal, or long-standing overt ventriculomegaly;
- Other approaches, i.e., endoscopic third ventriculostomy (ETV).

According to the exclusion criteria (see Section 2), 77 patients with possible and probable iNPH were initially included. To avoid potential confounding variables, a number of them were excluded: 3 patients with other types of hydrocephalus (like triventricular (1), tetraventricular (1) and fetal (1)), 8 patients who underwent other therapy approaches (like Lova (2), PSP (2), VCS (2), DVA (1), and radiotherapy (1)), 5 patients with other concomitant pathologies (such as neurinoma (2), AD (1), stroke (1), TCE (1)), 1 patient with addictions (potus), and 4 patients with incomplete datasets (cases n#14, n#26, n#45, and n#51).

The study was approved by the Ethics Committee “Comitato Etico Unico Regionale Fvg” (CEUR) (0043786/P/GEN/ARCS, ID 3456, Protocol No. 43201/Arcs; 9 December 2020) and carried out in accordance with the 2013 Fortaleza version of the Helsinki Declaration and subsequent amendments. Written informed consent was obtained from each participant.

The STROBE checklist was followed in reporting the study details.

### 2.2. Procedure

Patients underwent the Hydro-RTNT at their bedside, immediately before the infusion phase, at baseline (T0), at the end of the infusion phase, when the pressure plateau was reached (T1), and immediately at the end of the tap test, when the intracranial pressure was close to 0 mmHg (T2). The Hydro-RTNT approach does not alter the infusion and drainage of CSF procedure. Testing occurred independently of the tap test, as RTNT was added to a surgical procedure that was already being performed, without affecting the procedure itself. T0 and T2 testing were administered pre-tap test (when the patient was waiting to start the procedure) and post-tap test (after the procedure ended); thus, these phases are independent and do not require additional time. T1 testing occurs at the plateau; however, the duration of the test is negligible (a few minutes for backward counting; 1 min for verbal fluency; 1 min for naming plus letter detection).

**Figure 1:**
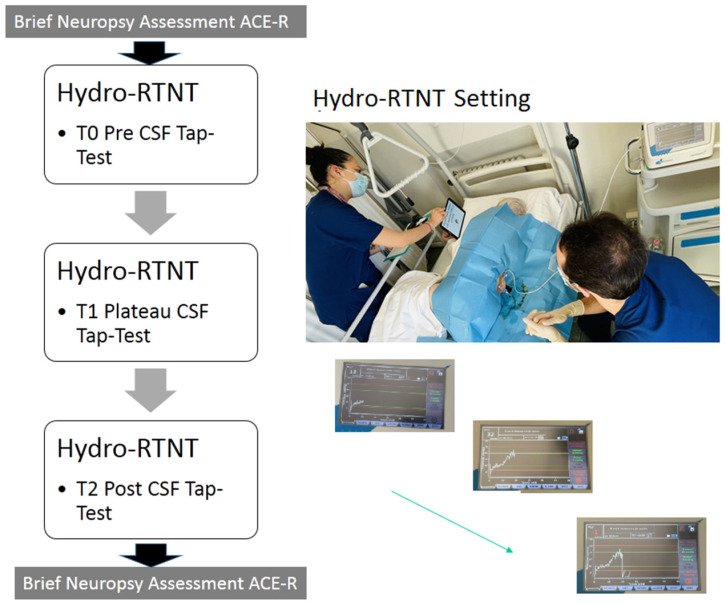
Study design and Hydro-RTNT setting.

Stimuli were presented on a tablet that could be easily brought close to the patient’s face (see Figure 1).

#### 2.2.1. Hydro-RTNT Protocol

Our experience with cognitive monitoring during awake surgery demonstrated that RTNT was versatile and adaptable to multiple settings and patients [19]. In clinical settings, when reliable neuropsychological information is needed, quick, short, and dynamic alternation of tests, i.e., RTNT, could be a useful approach. The Hydro-RTNT comprised a set of quick, rapid and reproducible tasks. The test lasted only a few minutes and was serviceable for old patients that were recumbent on their lateral position, during the infusion and tap tests. Visual stimuli were shown on a tablet.

The test protocol was created based on the clinical picture of patients with NPH, knowing that they can have impairments in executive functions, short-term memory/learning, psychomotor speed, and perceptual ability [12]. Therefore, the protocol included tests tapping impairments or improvements in these functions. These tests are standardized and normed for the Italian population (for their psychometric details, see the original publications). Parallel forms of tests can be assembled by neuropsychologists, using the standardized and normed tests for other populations. The protocol included the following:

- Backward counting test [23] requiring the patient to count backward from 20 to 1, for a total of 4 repetitions, to evaluate executive dysfunction in INPH, and forward counting from 1 to 20, for a total of 4 repetitions; in their original article [23], the authors excluded learning effects due to task repetition as the effect was not significant.
- Verbal fluency (one letter) [24] in 1 min. The patient is required to retrieve the maximum number of items following a key letter criterion. The key letter was randomized across T0, T1, and T2 presentations in order to rule out potential effects related to lexical frequency or learning.
- Naming of 5 action-related verbs [25]. The patient is presented with 5 pictures depicting an action and asked to produce the corresponding verb.
- Discrimination of 2 degraded letters [26]. The patient is presented with 2 figures representing a visually degraded letter each and is asked to detect which letter is shown. For the latter two tasks, stimuli changed every RTNT run, namely for T0, T1, and T2, so that learning effects could be avoided.

For backward and forward counting [23], we considered as measures the time taken to complete the task and accuracy. For the other tasks, we calculated the number of words produced on the verbal fluency task, the accuracy in the action-related verb naming task, and the total correct number of identified items on the discrimination task.

Following the authors’ instructions [23], we calculated (i) the first-error score, which is an average of the first-error numbers of the four trials, and (ii) the reverse effect index, which is the minimum completion time of the backward counting trial minus the minimum completion time of the forward counting trial. It was assumed [23] that the first-error score and the reverse effect index pointed to deficits in attention, deficits in working memory, and response suppression in patients with iNPH.

Time and space constraints account for the limited number of stimuli. During T1, the patient was in a lateral recumbent position, with the Tuohy needle inserted; a rapid and simple but efficient assessment was needed.

#### 2.2.2. Infusion and Tap Tests

The study of CSF dynamics was performed as previously reported [27,28]. With the patient lying in the lateral recumbent position, a Möller Medical LiquoGuard 7 (Fulda, Germany) pressure monitor and fluid infusion system was connected to a Tuohy spinal needle. CSF pressure was measured at baseline and during a saline solution infusion (at a constant rate of 1.5 mL/min) until a stable pressure plateau was reached. Resistance to outflow (ROUT) was calculated [21]. A cut-off of 12 mmHg/mL/min was used to differentiate between a positive and a negative test [21]. At the end of the infusion test, we performed a tap test, draining a quantity of CSF to reach a pressure of 0 mmHg.

#### 2.2.3. Brief Neuropsychological Assessment: Pre- and Post-Tap Test

Before the CSF infusion and tap test, patients underwent a brief neuropsychological assessment using Addenbrooke’s Cognitive Examination Revised (ACE-R) to determine their cognitive profiles. ACE-R evaluates attention and orientation (space and time); memory and learning skills; verbal fluency; verbal and written comprehension; naming, repetition, and writing and reading skills; and visual-perceptual and constructive skills. ACE-R is easy and quick to administer; it takes a maximum of 15 min [29]. After the tap test, when the physician said the patient’s head could be raised by 45–90°, the neuropsychologist re-administered the ACE-R (see Figure 1).

#### 2.2.4. Statistical Analysis

Descriptive statistical analyses were performed for all study variables. Neuropsychological data were not normally distributed, so nonparametric statistics was performed. For each single ACE-R test, we compared patients’ performance pre- and post-tap test by a Wilcoxon chi-square test, which allows the comparison of two related samples (Bonferroni correction for multiple comparisons).

Similarly, for each single Hydro-RTNT test, we compared patients’ performances at T1 vs. T0, T2 vs. T0, and T2 vs. T1 by the Friedman test, allowing the comparison of multiple related samples (Bonferroni correction for multiple comparisons). The relations between symptoms and cognitive performance and between infusion and tap test variables (pressure value at baseline and at a stable plateau and cognitive performance) were tested by means of Spearman correlation analyses. Data were analysed using SPSS 21.0 (SPSS, Inc., Chicago, IL, USA).

## 3. Results

### 3.1. Clinical Data

**Figure 2:**
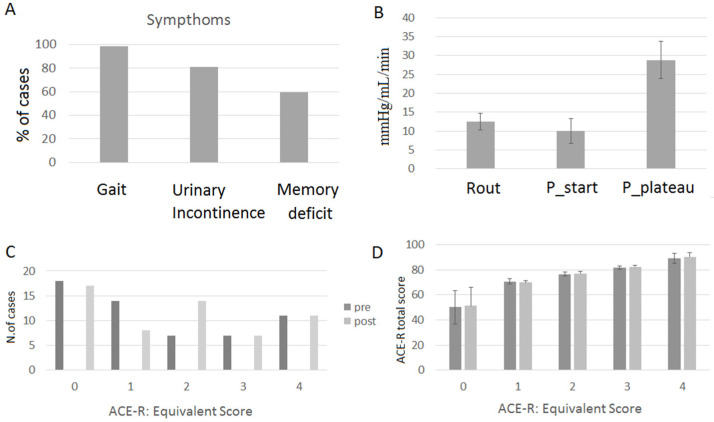
Patients’ triad of symptoms (**A**), CSF dynamics results (**B**), patients’ ACE-R equivalent scores (ranging from 0 = pathological, 1 = borderline, to 4) (**C**), and ACE-R total score pre- and post-CSF tap test (**D**). Error bars denote standard deviations.

The final analyses were carried out on a total of 57 patients (25 F, 32 M; mean age at referral 77.5 ± 5.8 years; mean education 10.1 ± 4.91 years; 55 right-handed, 2 ambidextrous) from the end of July 2020 to the end of December 2023. In the large majority of cases, patients presented gait impairment (Figure 2A). The symptom triad was complete in 30/57 cases.

### 3.2. Brief Neuropsychological Assessment: Pre-CSF Tap Test

The majority of the patients’ performances on the ACE-R were within the normal range (68.42%), with 31.57% of them obtaining an equivalent score (ES) of 0, which indicates cognitive impairment (Figure 2A). The ACE-R total score (maximum is 100) was high for patients with an ES of 1–4 (above 70/100, see Figure 2B) but not for patients with an ES of 0 (around 50/100, see Figure 2B).

### 3.3. CSF Dynamics Results

During the procedure, no complication was recorded. The mean ROUT value was 12.5 ± 2.23 mmHg/mL/min. Based on CSF dynamics, 36/57 (63.15%) patients showed high ROUT (>12 mmHg/mL/min values). Based on the tap test, 44/50 patients presented a clinical positive response. Their mean ROUT was 13.74 ± 1.35 mmHg/mL/min; patients with a ROUT < 12 mmHg/mL/min showed a mean of 10.274 ± 1.7 mmHg/mL/min.

Figure 2 shows the pressure values at baseline and at the end of the infusion phase, when a stable plateau was reached and the Hydro-RTNT was administered.

### 3.4. Hydro-RTNT Data

**Figure 3:**
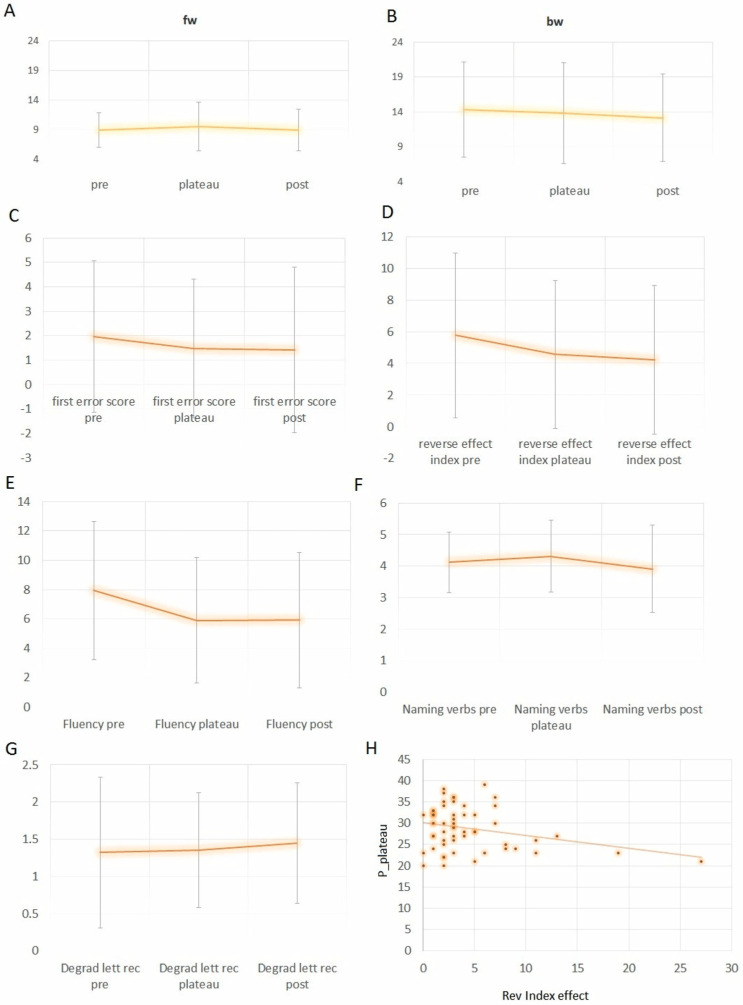
Results of the Hydro-RTNT. Mean completion time (seconds) of the forward (**A**) and backward (**B**) counting task and the first effort score (**C**) and the reverse index effect (**D**). Mean number of words produced on the verbal fluency task (**E**), mean number of correctly named actions (**F**) and correctly recognized degraded letters (**G**); correlation between P_plateau and reverse index effect (**H**). Error bars denote standard deviations.

The Friedman test determined whether scores differ between assessments. Significant effects were found for two executive function tests (backward counting and fluency test, Figure 3A–E). On backward counting, patients were significantly faster at T2 (vs. T1, Z = −2.97, *p* < 0.005; vs. T0, Z = −3.604, *p* < 0.001), and on forward counting, they were significantly faster at T2 vs. T1 (Z = −2.97, *p* < 0.005), suggesting an improvement in speed at T2 [Bonferroni correction α= 0.0083]. The reverse index significantly differed between measurements, χ^2^(2) = 13.74, *p* < 0.001 (Figure 3D); namely, there was a significantly smaller index at T1 vs. T0 (4.56 ± 4.66 vs. 5.77 ± 5.19, Z = −2.808 *p* = 0.005) and at T2 vs. T0 (4.22 ± 4.68 vs. 5.77 ± 5.19, Z = −3.604, *p* < 0.001), suggesting a more marked improvement in executive abilities at T2 and a smaller improvement at T1 [Bonferroni correction α = 0.0083]. The first error score did not differ, χ^2^(2) = 4.452, *p* = 0.108 (Figure 3C).

There was a significant difference in verbal fluency measurements, χ^2^(2) = 22.231, *p* < 0.001; namely, patients were worse at T1 vs. T0 (5.89 ± 4.28 vs. 7.92 ± 4.72 words, Z = −3.879 *p* < 0.001) and at T2 vs. T0 (5.92 ± 4.62 vs. 7.92 ± 4.72 words, Z = −3.705, *p* < 0.001), although the key letter was randomized between measurements (Figure 3E) [Bonferroni correction α = 0.016].

The other tests (action verb naming and degraded letter discrimination) did not significantly differ between measurements, χ^2^(2) = 5.314, *p* = 0.07 and χ^2^(2) = 4.395, *p* = 0.111 (Figure 3F,G).

### 3.5. Correlation Among Clinical and Cognitive Variables

There was a significant negative correlation between the reported memory deficit and patients’ fluency performance (r(56) = −0.422, *p* < 0.001) and a significant negative correlation between the pressure at the plateau and patients’ reverse index effect at T1 (r(56) = −0.283, *p* < 0.05) suggesting an increased severity of executive dysfunction with a lower pressure plateau value and a concurrently higher reverse index effect (see Figure 3H).

### 3.6. Brief Neuropsychological Assessment: Post-CSF Tap Test

The ACE-R administered post-procedure showed a significant change (χ^2^(16) 80.16, *p* < 0.001, [Bonferroni correction α = 0.0125]) in the number of patients with an ES of 1 (borderline performance) and an ES of 2, changing from 24.56% to 14.03% and from 12.28% to 24.56% pre-test vs. post-test, respectively. Overall, an improvement in the ACE-R total score was seen (Z= −2.509, *p* < 0.05; pre: 69.86 ± 16.67; post: 71.47 ± 16.79). A significant improvement in orientation was observed post-test vs. pre-test (Z= −2.509, *p* = 0.012 [Bonferroni correction α = 0.0125], pre: 8.29 ± 2.078; post: 8.7 ± 1.84).

## 4. Discussion

Based on our experience with RTNT in awake surgery, we tried to evaluate neuropsychological modifications, if any, during the infusion and tap test. The Hydro-RTNT approach differs from neuropsychological testing reported in the literature on iNPH patients (e.g., [15,16,17]) because it is an intra-procedure monitoring technique. In particular, neurocognitive assessment was used in previous studies only before or after the tap test [15,16,17]. Previous studies were aimed at detecting good predictors for a shunt intervention among test scores [17]. Here, we set out to assess the feasibility of an intra-procedure assessment. However, future studies are needed to establish whether the Hydro-RTNT test scores could serve as good predictors for a shunt intervention, also including the collection of post-shunt intervention data (which are missing in the present study). We believe the study goal was reached as we showed that fluctuations in cognitive performance could be detected at the end of the infusion phase and after CSF was removed. Fluctuations were not generalized, as they were selective for some of the tests administered. These tests were tests tapping executive functions, namely the backward counting task [23] and the verbal fluency task [25].

In addition, while previous studies showed cognitive amelioration between 30 and 60 min after the tap test [15], suggesting that testing carried out after the lumbar tap would provide much more reliable prognostic information, we showed that fluctuations in cognitive performance occur even earlier. We nonetheless acknowledge that, while the cortex mapping can quickly be performed during awake surgery for glioma, here, the cognitive improvement usually appears slowly after improvement of the intermittent intracranial hypertension.

We found that the reverse effect index was higher (i.e., high scores indicate a difference between backward and forward counting time, thus providing a measure of the iNPH patient’s executive abilities) at T0 vs. T1 and at T0 vs. T2. To interpret the reverse effect index, we looked at the time of completion and found that the reverse effect index at the plateau decreased because the time of completion for counting forward increased at the plateau. Patients were slower at counting forward at T1 vs. T2, indicating a worsening until a stable pressure plateau was reached, as compared to post-CSF drainage until pressure reached a value of 0 mmHg. Patients were slower at counting backward at T1 vs. T2, indicating a worsening after CSF drainage, as compared to a stable pressure plateau. Furthermore, they were slower at T0 than T2. Our results complement Kanno et al.’s [23] study showing that the backward counting task is useful for evaluating executive dysfunction in iNPH and show that this occurs also when the backward counting task is administered during the procedure. Authors argue that changes in the reverse effect index show the severity of executive dysfunction.

Second, we found that verbal fluency performance—it being a measure of executive functions, too—changed during the CSF tap test. We found a worsening at T1 (vs. T0) and at T2 (vs. T0). We ruled out the possibility that such a pattern was related to verbal frequency, as the key letter was randomized every time. We did not observe an improvement at T2; this raises the question of whether the T2 assessment was carried out at a proper time in order to observe significant changes. We performed the T2 testing right after the CSF drainage; however, clinical status and gait were evaluated later, 24h after the procedure. In future studies, we will address this issue by changing the timing of the Hydro-RTNT design. A re-evaluation of RTNT patients after 24 h could bypass the clinical condition of discomfort experienced by some patients at the end of the procedure, such as headache or an uncomfortable position (fully lying down). These aspects may have interfered with the levels of attention and concentration during task administration.

Some authors [30] studied the improvement in executive functions after shunt intervention in patients with NPH. Patients were administered tests tapping naming, short-term memory, attention, and fluency before and after the intervention and showed significant improvements. This shows that patients with NPH who respond positively to slow and continuous drainage of lumbar cerebrospinal fluid following ventriculoperitoneal shunting improve their performance on executive function tasks. Other authors [31], using the Computerized General Neuropsychological INPH Test (CoGNIT) on 41 patients, found an improvement after shunt in all cognitive domains assessed before surgery. Patients improved in memory, executive functions (Stroop test), attention, psychomotor speed, and manual dexterity. Overall, the observed pattern of performance on backward counting and verbal fluency tasks indicates that changes can be observed in real time in patients with iNPH due to CSF infusion. We thus suggest that the cognitive aspect may become a relevant index to consider in future infusion and tap tests. Such indices could be very informative, as it is known that even cognitive changes, if untreated, may likely become irreversible due to the pathogenesis of iNPH [32,33].

A third result concerns the difference in patients’ performance on the brief neuropsychological assessment, with ACE-R used pre- and post-procedure. This assessment is carried out at the patient’s bedside when the patient can be brought to a 45–90° position to facilitate both concentration and the administration of the copying and writing subtests. We found that patients obtaining an ES of 1 pre-procedure—1 indicating a borderline performance—obtained an ES of 2 indicating normal performance post-procedure. A consistent number of them showed this trend (12.28% pre-procedure to 24.56% post-procedure).

This might suggest that in patients with borderline performance, CSF removal leads to an improvement in performance, shifting it fully to normal. Subtests contributing raw scores to improvement include phonological and semantic fluency tests as well as memory tests, in line with what has been reported for domains most affected in patients with iNPH. This aspect is less evident in patients with pre-procedure performance largely in the normal range. However, it may be useful in the future to re-evaluate patients a few days after deliquoration to measure the long-term effectiveness of the procedure.

In general, we acknowledge that patients’ performances were quite inhomogeneous during evaluations; this was confirmed by the high standard deviations calculated for each task. In future studies with larger samples, it could be possible to stratify patients according to their initial pre-procedure brief neuropsychological assessment and test how they perform at the Hydro-RTNT.

This is a pilot study conducted to assess the feasibility of Hydro-RTNT in patients undergoing a tap test. In the future, the Hydro-RTNT could help surgeons to identify good responders to shunting producing positive motor effects, without causing negative cognitive and/or emotional changes. Such alterations, if not revealed in real time during the tap test, would likely be observed as post-surgical outcomes. We are planning to carry out a larger multicenter prospective study to demonstrate the role of neuropsychological modifications in iNPH patients. The Hydro-RTNT protocol could be easily adapted to different patients’ populations; parallel forms of tests can be assembled by neuropsychologists, using the standardized and normed tests for other populations.

### Limitations

This was the first study using a new, ultra-fast, and easy neuropsychological testing method for infusion and tap tests in patients with possible and probable iNPH. We acknowledge that our sample size is limited and could therefore limit the generalizability of the results. On the other hand, we used stringent inclusion and exclusion criteria in patients’ selection, and this could have reduced our eligible sample.

We also acknowledge that the postures used during this test may have influenced the effectiveness of cognitive tests. However, the neuropsychologist always ascertained that the patient could perform the test.

False positives and false negatives cannot be fully excluded, even when strategies to improve testing accuracy are used. Patients’ scores were evaluated in consideration of comprehensive clinical information collected from them. Performance was always evaluated as a relative change between serial neuropsychological testing, and not as single scores. We certainly need to identify better strategies to cope with false positives and false negatives when classifying single patients as good or bad responders in future studies.

Another limitation is that neuropsychological testing data after VPS implant are not presented, because the follow-up is still ongoing. For this reason, we cannot compare results at different times of follow-up. We are aware that the usefulness of the Hydro-RTNT approach can only be evaluated by testing putative correlations between intra-procedure and post-shunt data. This correlation analysis will also enable us to determine whether the Hydro-RTNT approach carries the same predictive power as currently established post-lumbar puncture tests. Finally, we assessed cognitive functions at the end of the tap test, but we cannot test patients after 12–24 h for logistic reasons. This is a problem that needs to be corrected in the future.

## 5. Conclusions

iNPH patients’ performance on executive function tests changed during procedure. The Hydro-RTNT protocol can contribute to improving the response of iNPH patients during the procedure in order to better select shunt-responsive patients in future approaches.

## Data Availability

The datasets analyzed for this study will be made available from the authors upon request. Data are not made publicly available owing to sensitive information that could affect the privacy of research participants.
